# Time Trends and Sociodemographic Factors Associated With Overweight and Obesity in Children and Adolescents in Spain

**DOI:** 10.1001/jamanetworkopen.2020.1171

**Published:** 2020-03-18

**Authors:** Jeroen de Bont, Yesika Díaz, Maribel Casas, Maria García-Gil, Martine Vrijheid, Talita Duarte-Salles

**Affiliations:** 1 Fundació Institut Universitari per a la recerca a l’Atenció Primària de Salut Jordi Gol i Gurina (IDIAPJGol), Barcelona, Spain; 2 Universitat Autònoma de Barcelona, Bellaterra, Spain; 3 ISGlobal, Barcelona, Spain; 4 Spanish Consortium for Research on Epidemiology and Public Health (CIBERESP), Madrid, Spain; 5 Universitat Pompeu Fabra, Barcelona, Spain

## Abstract

**Importance:**

Time-trend studies of overweight and obesity in childhood by sociodemographic factors are important for prioritizing public health initiatives. However, little is known about these trends in Spain, where high levels of obesity are found and where important demographic changes have occurred during the last 2 decades.

**Objective:**

To examine how time trends in the prevalence and incidence of overweight and obesity among children and adolescents differ by age, sex, socioeconomic status, urban/rural residence, and nationality.

**Design, Setting, and Participants:**

This cohort study included 1.1 million children and adolescents (aged 2-17 years) with at least 1 measure of height and weight in Catalonia, Spain, from 2006 to 2016. Electronic health records were accessed from the Information System for Research in Primary Care. Data analysis was conducted from January to December 2018.

**Main Outcomes and Measures:**

Prevalence and incidence rates and trends of overweight/obesity and obesity (overweight/obesity defined as having of BMI *z *score greater than 2.0 among children aged <5 years and greater than 1.0 among children aged ≥5 years; obesity defined as having of BMI *z *score greater than 3.0 among children aged <5 years and greater than 2.0 among children aged ≥5 years) between 2006 and 2016 were calculated and stratified by sociodemographic characteristics (ie, age, sex, deprivation index, urban/rural residence, and nationality).

**Results:**

The study population included 1 166 609 children and adolescents (570 982 [48.9%] girls; median [interquartile range] age at entry to electronic health record system, 2.4 [0-7.7] years; 1 006 892 [86.3%] with Spanish nationality). Of 941 041 children (80.7%) who lived in urban areas, 197 427 (20.7%) lived in the most deprived areas. Overall, the prevalence of overweight/obesity and obesity decreased between 2006 and 2016 in all sex and age groups; for example, among boys and girls aged 6 to 11 years, overweight/obesity prevalence decreased from 41.9% (95% CI, 41.5%-42.2%) to 39.9% (95% CI, 39.6%-40.3%) and from 39.7% (95% CI, 39.3%-40.2%) to 37.6% (95% CI, 37.3%-38.0%), respectively. Incidence rates of overweight/obesity and obesity were highest among children aged 6 to 7 years (overweight/obesity among boys: 11.9 [95% CI, 11.8-12.0] new cases per 100 person-years; obesity among boys: 4.9 [95% CI, 4.8-4.9] new cases per 100 person-years). Prevalence and incidence rates were highest in the most deprived areas, in urban areas, and among children with North, Central, or South American nationalities. Between 2006 and 2016, prevalence increased in the most deprived areas in almost all sex and age groups. Among girls aged 6 to 11 years living in the most deprived areas, the obesity prevalence ratio increased from 1.59 (95% CI, 1.46-1.74) to 2.03 (95% CI, 1.88-2.19) compared with those living in the least deprived areas. Furthermore, during this period, prevalence increased among children with non-Spanish nationalities, especially in the African and Asian nationality groups (eg, boys aged 6-11 years with Asian nationality compared with Spanish nationality, 2006: prevalence rate, 0.78 [95% CI, 0.60-1.01]; 2016: prevalence rate, 1.27 [95% CI, 1.15-1.39]). Incidence rates decreased among younger groups (eg, ≤7 years: incidence rate ratio for January 1, 2006, to June 30, 2011, vs July 1, 2011, to December 31, 2016: 0.94; 95% CI, 0.91-0.98) but remained stable in older groups.

**Conclusions and Relevance:**

In this study, the overall prevalence and incidence rates of childhood overweight/obesity and obesity slightly decreased during the last decade. However, increased deprivation disparities in childhood obesity were found, given that the prevalence increased among children living in deprived areas and with non-Spanish nationalities.

## Key Points

**Question:**

How do time trends in the prevalence and incidence of overweight and obesity among children and adolescents differ by sociodemographic factors?

**Findings:**

In this cohort study of more than 1.1 million children and adolescents, overall prevalence and incidence rates of childhood overweight/obesity and obesity slightly decreased during the last decade. However, prevalence increased among children from the most deprived areas and with non-Spanish nationalities, indicating increasing deprivation disparities.

**Meaning:**

In this study, despite an overall reduction in the prevalence of childhood overweight and obesity, increasing deprivation disparities were observed during the last decade.

## Teaser

This cohort study examines how time trends in the prevalence and incidence of overweight and obesity among children and adolescents in Spain differ by age, sex, socioeconomic status, urban/rural residence, and nationality.

## Introduction

The prevalence of overweight and obesity has plateaued in many developed countries during the last decade,^1,2^ but levels are still high. In Spain, approximately 41% of children aged between 6 and 9 years had overweight/obesity in 2015, the second highest prevalence in Europe.^3^ This prevalence is alarming given that childhood and adolescent obesity are associated with later life health consequences, including adult overweight/obesity^4^ and cardiovascular, musculoskeletal, and endocrine diseases.^5^

The prevalence of childhood and adolescent overweight/obesity in Europe is well described^3,6^ and is known to differ by sociodemographic characteristics such as age, sex,^7^ socioeconomic status (SES),^8^ rural/urban residence,^9^ and immigrant status.^10^ However, fewer studies have assessed how these sociodemographic factors are associated with variations in these time trends. Time-trend studies are important for monitoring and surveilling overweight/obesity rates as well as for planning future prevention strategies focused directly on population subgroups for whom trends are not reversing.^11^

Furthermore, information on incidence rates of overweight/obesity is still scarce because of the lack of longitudinal data. Age-specific incidence rates give information about the natural history of the development of overweight/obesity, identifying at which ages new cases are more likely to occur. To our knowledge, only a few studies have assessed incidence rates during childhood and adolescence, and they identified the highest incidence peak of obesity between the ages of 6 and 11 years.^12^ Thus, longitudinal data are needed to characterize obesity incidence by specific age groups, especially in Southern Europe, where the prevalence of obesity is high.^3^

In Spain, 2 sociodemographic processes have occurred since 2000. First, the 2008 economic crisis seriously affected Spain and led to increased socioeconomic inequality.^13^ Second, there was a large increase in immigration; between 2001 and 2016, the immigrant population in Catalonia increased from 3% to 14%.^14^ Increases in socioeconomic inequality and the size of the immigrant population are known to affect levels of overweight/obesity.^8,10^ Therefore, Spain is an interesting setting for the study of recent trends in the prevalence and incidence of childhood and adolescent overweight/obesity as well as the sociodemographic characteristics of these trends.

This study examined how time trends in the prevalence and incidence of overweight and obesity among children and adolescents differ by age, sex, SES, urban/rural residence, and nationality based on primary the health care data of more than 1.1 million children and adolescents in Catalonia, Spain.

## Methods

We used data from the Information System for Research in Primary Care (SIDIAP) in Catalonia, an autonomous community in the northeast of Spain that represents 16% of the Spanish population.^15^ The Information System for Research in Primary Care is a large deidentified electronic health record resource with longitudinal data starting in 2006. The SIDIAP population is highly representative of the Catalan region in terms of geographic area, age distribution, and sex distribution (representing approximately 80% of the Catalan population).^16^

In this cohort study, we included 1 166 609 children and adolescents between the ages of 2 and 17 years, registered in SIDIAP with at least 1 height and weight record during the same visit from January 1, 2006, to December 31, 2016 (eFigure 1 in the Supplement). Data were extracted from SIDIAP in 2017. This study was approved by the ethics committee of the Fundació Institut Universitari per a la recerca a l’Atenció Primària de Salut Jordi Gol i Gurina (IDIAPJGol). As SIDIAP is a deidentified database, the identification of individuals is not possible. Thus, consent was waived per the *International Ethical Guidelines for Epidemiological Studies*.^17^ We followed the Reporting of Studies Conducted Using Observational Routinely Collected Health Data (RECORD) statement.^18^

Body height and weight were routinely measured by pediatricians or pediatric nurses in primary care centers following the same protocol^19^ and used to calculate body mass index (BMI; calculated as weight in kilograms divided by height in meters squared). Age- and sex-specific BMI *z* scores (*z*BMI, in SD units) were calculated using the World Health Organization growth standard and growth reference.^20,21^ Biologically implausible values of height and weight were removed.^22,23^

Overweight and obesity were classified using the World Health Organization growth standard and reference. Children younger than 5 years with a *z*BMI greater than 2.0 and 3.0 were categorized as having overweight/obesity and obesity, respectively.^21^ Children aged 5 years and older with a *z*BMI greater than 1.0 and 2.0 were categorized as having overweight/obesity and obesity, respectively.^20^ Children not classified as overweight or obese were assigned to the normal weight category; this also included a small proportion of children with underweight (ie, *z*BMI <−2.0%; representing 4758 participants [0.4%]).^20,21^ Throughout this article, overweight including obesity is specified as overweight/obesity.

Information on sex, age, nationality, and municipality was obtained from SIDIAP. Information on SES was available through the Mortalidad en áreas pequeñas Españolas y desigualdades socio-económicas y ambientales (MEDEA) deprivation index, linked to each residential census area of the population.^24^ This deprivation index is based on 3 indicators related to work (ie, percentage of unemployment, percentage manual workers, and percentage eventual workers) and 2 indicators related to education (percentage with insufficient education [ie, unable to read and write or did not complete primary studies] overall and among young people) obtained from the Spanish national census of 2001.^24^ The deprivation index was only available for urban areas, defined as municipalities with more than 10 000 inhabitants and a population density greater than 150 habitants/km^2^; remaining areas were considered rural areas. We stratified the deprivation index in quintiles, in which the first and fifth quintiles were the least and most deprived, respectively. We used nationality as a proxy of immigrant status and grouped it in the 5 following categories: Spanish; African; North, Central, or South American; Asian; and European.

### Statistical Analysis

The prevalence and 95% CIs of overweight/obesity and obesity were calculated for age and study year. If a child had more than 1 BMI measurement during the same age and/or study year, the latest measurement was used. Prevalence was stratified by the following sociodemographic characteristics: sex, age (2-5 years, 6-11 years, and 12-17 years), deprivation index for urban areas in quintiles, urban/rural residence, and nationality (5 categories). Prevalence trends over time were evaluated with logistic regression models using the study year as a continuous variable (ie, independent variable) and overweight/obesity as binary variables (ie, dependent variables). To estimate differences in prevalence by sociodemographic characteristics, the prevalence ratio (PR) was calculated with a Poisson regression with robust variance. Furthermore, we calculated the percentage change of prevalence over time in categories of sociodemographic characteristics by dividing the difference between the prevalence in 2016 and the prevalence in 2006 by the prevalence in 2006 and multiplying by 100.

Incidence rates were estimated for children who had at least 2 BMI measurements and were not overweight or obese at baseline (ie, at first BMI measurement). The incidence rate was calculated by dividing the number of new cases of overweight/obesity or obesity by 100 person-years of follow-up. Person-years were calculated as the years at risk of developing overweight/obesity or obesity during a specific age period. Age periods were considered for consecutive years (age 2-3 years, 4-5 years, 6-7 years, 8-9 years, 10-11 years, 12-13 years, 14-15 years, and 16-17 years) to balance the number of observations, which were higher at even-numbered ages. Children were observed until they reached age 18 years, transferred, or died or until the study period ended (ie, December 31, 2016). Incidence rates were stratified by sociodemographic characteristics, and incidence rate ratios were calculated to compare the differences in incidence between sociodemographic characteristics. Furthermore, the risk of becoming obese was compared with having normal weight or overweight (not including obesity) at baseline. To assess the incidence trend over time, we calculated the incidence in the 2 following periods: January 1, 2006, to June 30, 2011, and July 1, 2011, to December 31, 2016. It was not possible to assess the incidence by period for deprivation, urban/rural residence, or nationality because of small sample size and confidentiality issues. Statistical significance was set at *P* < .05, and all tests were 2-tailed. Analyses were conducted in R Core Team 2016 (R Project for Statistical Computing). Data analysis was performed from January to December 2018.

## Results

The study population included 1 166 609 children and adolescents with 4 167 703 valid BMI measurements (eFigure 1 in the Supplement). Among the study population, 570 982 children (48.9%) were girls, 941 041 (80.7%) lived in urban areas, and 1 006 892 (86.3%) had Spanish nationality (Table 1). During the study period, the number of BMI measurements increased among children living in the most deprived urban areas (2006, 48 508 of 247 669 [19.6%]; 2016, 70 820 of 337 291 [21.0%]). The number of children with non-Spanish nationality increased during the during period from 18 399 (6.6%) in 2006 to 45 503 (12.7%) in 2016. More immigrants lived in the most deprived urban areas than the least deprived urban areas (44 033 of 194 426 [22.6%] vs 13 402 of 154 915 [8.7%]). Of 34 536 children (3.0%) with North, Central, or South American nationalities, 33 977 (98.4%) had Central or South American nationality, including Mexico. The median (interquartile) age when entering SIDIAP was 2.4 (0-7.7) years. Of the children living in urban areas, 197 427 (20.7%) were living in the most deprived areas. Compared with 744 966 children (63.9%) classified has having normal weight, more children of 175 144 (15.0%) classified as having obesity in at least 1 BMI measurement were boys (373 560 [50.1%] vs 101 979 [58.2%]), lived in more deprived areas (119 563 [20.0%] vs 34 552 [23.9%]) or urbanized areas (596 559 [80.1%] vs 144 377 [82.4%]), and had longer median (interquartile range) follow-up time (4.9 [2.3-7.9] years vs 7.8 [5.3-9.9] years) (Table 1).

**Table 1.** Population Characteristics of 1 166 609 Children and Adolescents From the SIDIAP Database, 2006 to 2016

Characteristic	Children, No. (%)			
	Total (N = 1 166 609)	With normal weight (n = 744 966)^a^	With overweight (n = 246 499)^b^	With obesity (n = 175 144)^c^
Age entering SIDIAP, median (IQR), y	2.4 (0.0-7.7)	1.3 (0.0-7.2)	3.9 (0.1-8.4)	3.8 (0.3-7.8)
Girls	570 982 (48.9)	373 394 (50.1)	124 423 (50.5)	73 165 (41.8)
Follow-up time after first BMI measurement, median (IQR), y	5.9 (3.2-8.8)	4.9 (2.3-7.9)	7.1 (4.6-9.5)	7.8 (5.3-9.8)
BMI measurement, median (IQR)	3 (2-5)	2 (1-4)	3 (2-5)	5 (3-7)
Deprivation index, quintile^d^				
First, least deprived	154 915 (16.5)	104 771 (17.6)	32 519 (16.3)	17 625 (12.2)
Second	170 532 (18.1)	108 987 (18.3)	37 074 (18.5)	24 471 (16.9)
Third	174 748 (18.6)	108 457 (18.2)	38 200 (19.1)	28 091 (19.5)
Fourth	179 838 (19.1)	109 886 (18.4)	38 962 (19.5)	30 990 (21.5)
Fifth, most deprived	194 426 (20.7)	119 563 (20.0)	40 311 (20.1)	34 552 (23.9)
Missing	66 582 (7.1)	44 895 (7.5)	13 039 (6.5)	8648 (6.0)
Urban/rural residence				
Rural	225 568 (19.3)	148 407 (19.9)	46 394 (18.8)	30 767 (17.6)
Urban	941 041 (80.7)	596 559 (80.1)	200 105 (81.2)	144 377 (82.4)
Nationality				
Spanish	1 006 892 (86.3)	631 084 (85.3)	216 812 (88.0)	155 489 (88.8)
African	64 312 (5.5)	47 757 (6.5)	10 407 (4.2)	5687 (3.2)
North, Central, or South American	34 536 (3.0)	18 952 (2.6)	8692 (3.5)	6761 (3.9)
Asian	26 021 (2.2)	18 266 (2.5)	4263 (1.7)	3052 (1.7)
European	34 848 (3.0)	24 149 (3.3)	6325 (2.6)	4155 (2.4)

Abbreviations: BMI, body mass index (calculated as weight in kilograms divided by height in meters squared); IQR, interquartile range; SIDIAP, Information System for Research in Primary Care.

^a^ Children were categorized as having normal weight if they were never categorized as having overweight or obesity.

^b^ Children were categorized as having overweight/obesity if they had at least 1 age- and sex-specific BMI *z *score classified as having overweight or any measurement classified as having obesity during the study period. Children younger than 5 years were categorized as having overweight if their BMI was at least 2.0 SD units greater than age-specific BMI *z *scores; children aged 5 years and older were categorized as having overweight if their BMI was at least 1.0 SD unit greater than age-specific BMI *z* scores.

^c^ Children were categorized as having obesity if they had at least 1 age- and sex-specific BMI *z *score classified as having obesity during the study period. Children younger than 5 years were categorized as having obesity if their BMI was at least 3.0 SD units greater than age-specific BMI *z *scores; children aged 5 years and older were categorized as having overweight if their BMI was at least 2.0 SD units greater than age-specific BMI *z *scores.

^d^ Deprivation index only available for 941 041 children (80.7%) living in urban areas.

### Prevalence of Overweight and Obesity

The overall prevalence of overweight/obesity was the highest between the ages of 6 and 11 years (boys: 44.9%; 95% CI, 44.7%-45.0%; girls: 41.8%; 95% CI, 41.6%-41.9%) and the lowest between the ages of 2 and 5 years (boys: 14.6%; 95% CI, 14.5%-14.8%; girls: 13.7%; 95% CI, 13.6%-13.9%) (Table 2). The prevalence of obesity followed the same pattern and was 21.7% (95% CI, 21.6%-21.9%) among boys aged 6 to 11 years and 16.3% (95% CI, 16.2%-16.4%) among girls aged 6 to 11 years. The most deprived urban areas had a higher prevalence of overweight/obesity and obesity compared with the least deprived areas for all age-sex categories (Table 2). The prevalence of obesity among children aged 6 to 11 years living in the most deprived urban areas was 25.1% (95% CI, 24.8%-25.5%) among boys and 19.9% (95% CI, 19.6%-20.2%) among girls, whereas it was 17.0% (95% CI, 16.7%-17.4%) among boys and 11.9% (95% CI, 11.6%-12.2%) among girls living in the least deprived areas (Table 2). Children living in urban areas and with North, Central, or South American nationalities had a higher prevalence of overweight/obesity and obesity than children living in rural areas and with other nationalities in all age and sex categories (eg, girls aged 6-11 years in urban vs rural areas: 16.7% [95% CI, 16.6%-16.9%] vs 14.6% [95% CI, 14.4%-14.9%]; with North, Central, or South American nationality vs Spanish nationality: 21.5% [95% CI, 20.7%-22.4%] vs 16.6% [95% CI, 16.5%-16.7%]) (Table 2).

**Table 2.** Prevalence of Overweight/Obesity and Obesity by Sex, Age, Deprivation Index, Urban/Rural Residence, and Nationality, 2006 to 2016

Characteristic	Prevalence by age, % (95% CI)					
	Overweight/obesity^a^			Obesity^b^		
	2-5 y	6-11 y	12-17 y	2-5 y	6-11 y	12-17 y
**Boys**						
Overall						
2006-2016	14.6 (14.5-14.8)	44.9 (44.7-45.0)	39.9 (39.7-40.1)	4.1 (4.1-4.2)	21.7 (21.6-21.9)	15.7 (15.6-15.9)
2006	11.2 (10.9-11.4)	41.9 (41.5-42.2)	38.5 (37.9-39.1)	3.3 (3.2-3.5)	20.1 (19.8-20.5)	15.4 (15.0-15.9)
2016	8.6 (8.3-8.8)	39.9 (39.6-40.3)	36.7 (36.3-37.2)	2.9 (2.7-3.0)	19.6 (19.3-19.9)	14.3 (14.0-14.6)
Deprivation index, quintile						
First, least deprived	13.1 (12.8-13.4)	40.7 (40.3-41.2)	35.5 (35.0-36.1)	3.7 (3.5-3.9)	17.0 (16.7-17.4)	11.7 (11.4-12.1)
Second	13.9 (13.6-14.2)	45.0 (44.6-45.4)	39.7 (39.2-40.3)	4.4 (4.2-4.5)	21.2 (20.8-21.5)	14.9 (14.6-15.3)
Third	15.0 (14.7-15.3)	47.1 (46.7-47.5)	41.8 (41.3-42.3)	5.1 (4.9-5.3)	23.4 (23.1-23.8)	16.6 (16.2-17.0)
Fourth	15.9 (15.6-16.2)	47.9 (47.5-48.4)	43.0 (42.5-43.5)	5.5 (5.4-5.7)	24.9 (24.5-25.2)	17.9 (17.5-18.3)
Fifth, most deprived	16.9 (16.6-17.2)	47.2 (46.8-47.6)	42.1 (41.6-42.5)	6.3 (6.2-6.5)	25.1 (24.8-25.5)	18.8 (18.5-19.2)
Urban/rural residence						
Rural	13.4 (13.2-13.7)	41.7 (41.4-42.1)	37.3 (36.9-37.8)	4.1 (4.0-4.3)	19.0 (18.7-19.3)	14.1 (13.8-14.4)
Urban	15.0 (14.8-15.1)	45.6 (45.5-45.8)	40.5 (40.3-40.7)	5.0 (4.9-5.1)	22.4 (22.3-22.6)	16.1 (15.9-16.3)
Nationality						
Spanish	14.6 (14.5-14.7)	45.6 (45.5-45.8)	40.9 (40.7-41.1)	4.8 (4.7-4.9)	22.1 (22.0-22.3)	16.1 (16.0-16.3)
African	13.9 (13.5-14.4)	29.7 (29.0-30.4)	20.5 (19.7-21.3)	4.1 (3.8-4.4)	12.4 (11.9-12.9)	6.4 (5.9-6.9)
North, Central, or South American	22.5 (21.4-23.7)	55.6 (54.6-56.6)	43.5 (42.5-44.6)	8.6 (7.9-9.4)	29.5 (28.5-30.5)	18.4 (17.6-19.2)
Asian	14.8 (14.0-15.6)	41.2 (40.0-42.3)	31.8 (30.5-33.1)	6.4 (5.9-7.0)	21.5 (20.5-22.5)	12.4 (11.5-13.4)
European	12.7 (12.1-13.4)	38.5 (37.5-39.4)	36.5 (35.3-37.8)	4.2 (3.8-4.6)	17.9 (17.1-18.6)	14.7 (13.8-15.6)
**Girls**						
Overall						
2006-2016	13.7 (13.6-13.9)	41.8 (41.6-41.9)	34.0 (33.8-34.2)	4.1 (4.0-4.2)	16.3 (16.2-16.4)	10.5 (10.4-10.6)
2006	10.8 (10.5-11.0)	39.7 (39.3-40.2)	32.1 (31.5-32.7)	2.9 (2.8-3.1)	15.3 (15.0-15.6)	9.9 (9.6-10.3)
2016	8.6 (8.3-8.8)	37.6 (37.3-38.0)	31.7 (31.3-32.1)	2.5 (2.3-2.6)	14.7 (14.5-15.0)	9.9 (9.6-10.1)
Deprivation index						
First	12.0 (11.7-12.3)	37.2 (36.7-37.6)	27.8 (27.3-28.3)	3.0 (2.8-3.2)	11.9 (11.6-12.2)	6.9 (6.6-7.1)
Second	12.9 (12.6-13.2)	41.4 (41.0-41.8)	32.7 (32.2-33.2)	3.6 (3.4-3.8)	15.1 (14.8-15.4)	9.3 (9.0-9.6)
Third	14.2 (13.9-14.5)	43.6 (43.2-44.0)	35.3 (34.8-35.8)	4.3 (4.2-4.5)	17.2 (16.9-17.5)	11.0 (10.6-11.3)
Fourth	15.1 (14.8-15.4)	45.4 (45.0-45.9)	37.5 (37.0-38.0)	4.9 (4.7-5.1)	19.0 (18.7-19.3)	12.2 (11.8-12.5)
Fifth	15.9 (15.6-16.2)	44.9 (44.4-45.3)	38.8 (38.3-39.3)	5.3 (5.1-5.5)	19.9 (19.6-20.2)	13.8 (13.4-14.1)
Urban/rural residence						
Rural	12.6 (12.3-12.8)	38.8 (38.4-39.2)	31.7 (31.2-32.1)	3.6 (3.5-3.7)	14.6 (14.4-14.9)	9.7 (9.4-9.9)
Urban	14.0 (13.9-14.2)	42.5 (42.3-42.7)	34.5 (34.3-34.8)	4.2 (4.2-4.3)	16.7 (16.6-16.9)	10.7 (10.6-10.9)
Nationality						
Spanish	13.8 (13.7-13.9)	42.5 (42.3-42.6)	33.8 (33.6-34.1)	4.1 (4.1-4.2)	16.6 (16.5-16.7)	10.5 (10.3-10.6)
African	12.8 (12.3-13.2)	31.9 (31.2-32.6)	32.7 (31.7-33.7)	3.5 (3.2-3.7)	11.5 (11.0-12.0)	9.7 (9.1-10.3)
North, Central, or South American	21.2 (20.1-22.3)	50.9 (49.9-52.0)	43.0 (42.0-44.0)	7.6 (6.9-8.3)	21.5 (20.7-22.4)	14.2 (13.5-14.9)
Asian	10.9 (10.1-11.6)	31.2 (30.0-32.4)	27.3 (25.9-28.7)	3.2 (2.7-3.6)	10.3 (9.5-11.0)	8.0 (7.2-8.9)
European	11.4 (10.7-12.0)	34.2 (33.2-35.1)	30.8 (29.7-32.0)	3.6 (3.2-3.9)	12.7 (12.0-13.4)	9.0 (8.3-9.7)

^a^ Children were categorized as having overweight/obesity if they had at least 1 age- and sex-specific body mass index (BMI) *z *score classified as having overweight or any measurement classified as having obesity during a specific age category (2-5, 6-11, or 12-17 years). Children younger than 5 years were categorized as having overweight if their BMI was at least 2.0 SD units greater than age-specific BMI *z *scores; children aged 5 years and older were categorized as having overweight if their BMI was at least 1.0 SD unit greater than age-specific BMI *z* scores.

^b^ Children were categorized as having obesity if they had at least 1 age- and sex-specific BMI *z *score classified as having obesity during a specific age category (2-5, 6-11, or 12-17 years). Children younger than 5 years were categorized as having obesity if their BMI was at least 3.0 SD units greater than age-specific BMI *z *scores; children aged 5 years and older were categorized as having overweight if their BMI was at least 2.0 SD units greater than age-specific BMI *z *scores.

### Trends in the Prevalence of Overweight and Obesity

Overall, the prevalence of overweight/obesity and obesity decreased between 2006 and 2016 for all age-sex categories (eFigure 2 in the Supplement). For example, among children aged 6 to 11 years, the prevalence of overweight/obesity decreased from 41.9% (95% CI, 41.5%-42.2%) to 39.9% (95% CI, 39.6%-40.3%) among boys and from 39.7% (95% CI, 39.3%-40.2%) to 37.6% (95% CI, 37.3%-38.0%) among girls (Table 2). However, an increase in PR of overweight/obesity was observed among children aged 6 to 11 years and 12 to 17 years living in the most deprived areas (6-11 years, 2006: PR, 1.15 [95% CI, 1.09-1.21]; 2016: PR, 1.30 [95% CI, 1.24-1.35]; 12-17 years, 2006: PR, 1.07 [95% CI, 1.00-1.15]; 2016: PR, 1.33 [95% CI, 1.25-1.41]) (Table 3; eFigure 3 in the Supplement). In all age-sex groups except among girls aged 12 to 17 years, the prevalence of overweight/obesity and obesity decreased more quickly in the least deprived compared with the most deprived areas, indicating increased inequality between 2006 and 2016 (ie, higher PRs). For example, in 2006, the PR of obesity among boys aged 6 to 11 years living in the most deprived areas compared with those living in the least deprived areas was 1.39 (95% CI, 1.29-1.50); in 2016, this increased to 1.79 (95% CI, 1.68-1.90). In girls of that age, the obesity PR for the most vs the least deprived areas increased from 1.59 (95% CI, 1.46-1.74) to 2.03 (95% CI, 1.88-2.19) (Table 3).

**Table 3.** Prevalence Trends and Ratios for Overweight/Obesity and Obesity by Sex, Age, and Deprivation Index, 2006 to 2016

Age, y	Deprivation index	Overweight/obesity				Obesity			
		Prevalence ratio (95% CI)^a^		Change from 2006-2016, %^b^	*P* value for trend^c^	Prevalence ratio (95% CI)^a^		Change from 2006-2016, %^b^	*P* value for trend^c^
		2006	2016			2006	2016		
**Boys**									
2-5	Least deprived	1 [Reference]	1 [Reference]	−26.81	<.001	1 [Reference]	1 [Reference]	−20.41	<.001
	Most deprived	1.39 (1.27-1.53)	1.55 (1.39-1.72)	−18.49	<.001	1.82 (1.52-2.16)	2.08 (1.72-2.52)	−8.93	<.001
6-11	Least deprived	1 [Reference]	1 [Reference]	−10.35	<.001	1 [Reference]	1 [Reference]	−17.10	<.001
	Most deprived	1.15 (1.09-1.21)	1.30 (1.24-1.35)	1.04	.44	1.39 (1.29-1.50)	1.79 (1.68-1.90)	6.40	.37
12-17	Least deprived	1 [Reference]	1 [Reference]	−15.34	<.001	1 [Reference]	1 [Reference]	−21.99	<.001
	Most deprived	1.07 (1.00-1.15)	1.33 (1.25-1.41)	4.87	.57	1.45 (1.29-1.63)	1.91 (1.73-2.11)	2.92	.85
**Girls**									
2-5	Least deprived	1 [Reference]	1 [Reference]	−28.24	<.001	1 [Reference]	1 [Reference]	−37.40	<.001
	Most deprived	1.34 (1.22-1.48)	1.54 (1.38-1.73)	−17.55	<.001	1.66 (1.37-2.02)	2.38 (1.88-3.01)	−10.46	.02
6-11	Least deprived	1 [Reference]	1 [Reference]	−10.66	<.001	1 [Reference]	1 [Reference]	−15.86	<.001
	Most deprived	1.18 (1.12-1.25)	1.34 (1.28-1.40)	0.91	.21	1.59 (1.46-1.74)	2.03 (1.88-2.19)	7.26	<.001
12-17	Least deprived	1 [Reference]	1 [Reference]	−3.21	<.001	1 [Reference]	1 [Reference]	4.88	.08
	Most deprived	1.39 (1.28-1.50)	1.48 (1.39-1.58)	3.28	.21	2.22 (1.89-2.62)	2.24 (1.98-2.54)	5.82	.03

^a^ Prevalence ratios were calculated using a Poisson regression with robust variance.

^b^ The percentage change of prevalence from 2006 to 2016 was calculated by dividing the difference between the prevalence in 2016 and 2006 by the prevalence in 2006 and multiplying by 100.

^c^ Logistic regression models were applied to test the *P* value for trend using the study year as a continuous variable.

Prevalence of overweight/obesity and obesity decreased between 2006 and 2016 in rural and urban areas across almost all age-sex groups, and somewhat larger decreases were observed in rural areas, indicating slightly greater urban-rural differences (ie, higher PRs) in 2016 compared with 2006 (eg, overweight/obesity among boys aged 6-11 years, 1.14 [95% CI, 1.10-1.17] vs 1.09 [95% CI, 1.05-1.12]) (eTable 1 in the Supplement).

The decreasing trends in overweight/obesity and obesity observed in the overall population were not seen in the non-Spanish nationality categories across most age-sex groups (eTable 2 and eFigure 4 in the Supplement). Indeed, increasing PRs were observed among children with non-Spanish nationalities compared with those with Spanish nationality; childhood overweight/obesity prevalence in non-Spanish groups, especially among children with African and Asian nationalities, were approaching those among children with Spanish nationality. For example, comparing Asian nationality with Spanish nationality, the obesity PR among boys aged 6 to 11 years increased from 0.78 (95% CI, 0.60-1.01) in 2006 to 1.27 (95% CI, 1.15-1.39) in 2016 (eTable 2 and eFigure 4 in the Supplement).

### Incidence of Overweight and Obesity

The incidence of overweight/obesity and obesity were highest among children aged 6 to 7 years. The number of new overweight/obesity cases per 100 person-years was 11.9 (95% CI, 11.8-12.0) among boys and 11.2 (95% CI, 11.1-11.3) among girls, and the number of new obesity cases per 100 person-years was 4.9 (95% CI, 4.8-4.9) among boys and 3.9 (95% CI, 3.8-3.9) among girls (Figure; eTable 3 in the Supplement). Incidence rates decreased among children aged 8 to 17 years. Children who were overweight at baseline had higher incidence rates of obesity (boys: 12.1 [95% CI, 12.0-12.4] new cases of obesity per 100 person-years; girls: 11.2 [95% CI, 10.9-11.4] new cases of obesity per 100 person-years) than children who had normal weight at baseline (boys: 4.1 [95% CI, 4.1-4.2] new cases of obesity per 100 person-years; girls: 3.1 [95% CI, 3.0-3.2] new cases of obesity per 100 person-years) (eFigure 5 in the Supplement). Overweight/obesity and obesity incidence rates were higher among children living in the most deprived areas compared with those living in the least deprived areas (eg, overweight/obesity among boys aged 6-7 years: 1.21 [95% CI, 1.17-1.26]) and in urban areas compared with rural areas (eg, overweight/obesity among boys aged 6-7 years: 1.13 [95% CI, 1.10-1.15]) (eTable 4 and eTable 5 in the Supplement). Children of North, Central, and South American nationalities had higher incidence rates of overweight/obesity and obesity than children with other nationalities (eg, boys aged 6-7 years with North, Central, or South American nationality: 1.23 [95% CI, 1.14-1.33]; with African nationality: 0.75 [95% CI, 0.72-0.79]) (eTable 4 and eTable 5 in the Supplement). The incidence of overweight/obesity and obesity decreased significantly (ie, incidence rate ratio <1) between period 1 (ie, January 1, 2006, to June 30, 2011) and period 2 (ie, July 1, 2011, to December 31, 2016) among children younger than 7 years. For example, the obesity incidence rate ratio was 0.94 (95% CI, 0.91-0.98) among boys aged 6 to 7 years in period 2 compared with period 1. At older ages, the incidence rate ratios did not show differences between the 2 periods (eTable 4 and eTable 5 in the Supplement).

**Figure.**
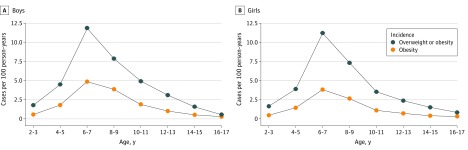
Incidence of Overweight/Obesity and Obesity by Sex Whiskers represent 95% CIs.

## Discussion

This cohort study of more than 1.1 million children and adolescents found that, despite a slight overall decrease in the prevalence of overweight/obesity and obesity between 2006 and 2016, disparities between groups based on level of SES deprivation increased in urban areas. The incidence rates of overweight/obesity and obesity were highest among children aged 6 to 7 years, and they decreased over time among younger groups (ie, ≤7 years) but remained stable in older groups. Children living in urban areas had higher prevalence and incidence rates of overweight/obesity and obesity than children living in rural areas. Children with North, Central, or South American nationalities showed the highest prevalence and incidence rates. Prevalence increased substantially among children with African and Asian nationalities between 2006 and 2016.

The trend of overweight/obesity and obesity has plateaued or slightly decreased in many developed countries during the 21st century.^1^ In Spain, a trend analysis by the national health survey found no significant differences in the prevalence of childhood and adolescent overweight/obesity and obesity between 2006 and 2011.^2^ In our study, prevalence rates were similar, as they were in other representative Spanish studies.^25,26^ The Catalan region is similar to rest of the Spain in terms of age, sex, and nationality distributions.^27^ Although Catalonia is among the wealthiest areas of Spain, similar socioeconomic trends in preventable mortality are found in all Spanish urban areas, indicating possible similar inequalities across Spain.^28^ Furthermore, in this study, we found a significant overall decrease of overweight/obesity and obesity already between 2006 and 2011 compared with a previous study.^2^ This may be explained by our larger sample size and our more reliable height and weight measurements, which were performed by health professionals rather than the self-reports used in the other study.^2^

A 2016 systematic review has shown different trends of childhood overweight/obesity and obesity prevalence by SES in high-income countries.^29^ The systematic review showed that approximately 40% of the studies have shown a widening of the socioeconomic inequality gap since 2000, while the rest of the studies did not find changes or found a narrowing of the inequality gap. Studies were conducted mainly in the US, Australia, France, Sweden, and Wales, which have a lower prevalence of obesity.^3^ Most of these studies were based on health surveys with limited age categories and in which stratification by sex was not always possible. Our study showed that, in urban areas across a broad age range in both sexes, the prevalence of overweight/obesity and obesity was decreasing in the least deprived areas but stabilizing or even increasing in the most deprived areas. This indicates an increase in the inequality gap. The reasons for this increase are not clear, although we may give some possible explanations. First, the 2008 economic crisis increased socioeconomic inequality in Spain, increasing the size of disadvantaged populations in the most deprived areas.^13^ These populations are more likely to have unhealthy nutritional habits, which may lead to increased obesity levels.^13^ Second, since the beginning of the 21st century, there has been an increase in residential segregation in Catalonia,^30^ increasing the number of people living in areas at the extremes of the socioeconomic gradient. In fact, our data showed an increase in the number of BMI observations in the least and most deprived areas between 2006 and 2016. More deprived areas are more exposed to poverty, crime, low social cohesion, lack of green spaces, higher levels of air pollution, and poor quality of the built environment, which could increase childhood obesity.^31^ Finally, the proportion of individuals with non-Spanish nationalities in SIDIAP increased from 9% in 2006 to 13% in 2016. We observed that children with non-Spanish nationality had a lower prevalence of overweight/obesity and obesity at the start of the study period, but their risk of overweight/obesity and obesity increased over time, in line with increasing duration of residence in Spain, especially among children with African and Asian nationalities. This trend has been seen in other studies, in which exposure and acculturation to the Western lifestyle among immigrants were thought to lead to this increase.^32^ However, information on childhood overweight and obesity in immigrants in Europe and Spain is scarce and may differ substantially from what is reported in the US. Following our results, we conclude that immigration patterns may play an important role in the socioeconomic inequalities in trends of overweight and obesity in urban areas in Spain, given that most immigrants tend to live in urban areas and in the most deprived areas.

Understanding the period during which the first cases of overweight/obesity and obesity are most likely to occur is essential for targeting health initiatives at specific age groups, but to our knowledge, only a few studies developed in the US, England, and Germany^33,34,35^ have assessed the incidence rate of overweight/obesity and obesity during childhood and adolescence.^12^ These studies found the highest incidence rates during midchildhood (ie, age 6-11 years), which decreased among older groups, except for the German study,^34^ which found similar incidence levels between age 2 and 6 years.^33,34,35^ Similar results were found in our study, in which the highest peak of obesity incidence was found between ages 6 and 7 years and decreased at older ages. This decrease could be explained by a decrease in children at risk or by growth and development changes, such as the adiposity rebound and the body composition changes that take place during puberty, which decrease the risk of developing overweight/obesity or obesity.^12^ Furthermore, we found that children with overweight were more likely to have obesity later in life than children with normal weight across all age groups. In the US study,^33^ children with overweight had 4 times more risk of having obesity among both sexes. Similarly, in our study, we found that overweight boys and girls had 2.9 and 3.6 times higher incidence rates of obesity between age 6 and 7 years, respectively, compared with children with normal weight. These results highlight the importance of public health promotion programs at early ages, during which primary health care professionals can play a key role identifying children with overweight during routine visits.

Geographic variation exists in the prevalence of overweight/obesity and obesity between rural and urban areas across the globe. In the US^9^ and Norway,^36^ children living in rural areas have a higher prevalence of obesity, whereas in other countries, including China^37^ and New Zealand,^38^ the opposite is true. Previous studies in Spain have shown that children living in urban areas are less physically active, have lower adherence to the Mediterranean diet, and eat more high-caloric meals than children living in rural areas,^39,40^ which could lead to higher obesity levels. In our study, rural areas tended to have more children with Spanish nationality than urban areas (ie, 90% in rural areas vs 85% in urban areas), but children living in rural and urban areas were similar in age and sex. Future studies are needed to evaluate the possible mechanisms on the observed rural-urban differences.

Regarding variation by nationality, the prevalence of overweight/obesity and obesity was the highest among children with North, Central, or South American nationality and the lowest among children with African nationality. Previous studies have shown that residents of Central and South America have among the highest prevalence rates of overweight/obesity and obesity in the world (98.4% of our sample of individuals with North, Central, or South American nationality were from Central or South America), while children with African nationality have among the lowest prevalence rates.^10^

### Strengths and Limitations

The main strengths of this study are its large sample size and the availability of repeated weight and height records across childhood and adolescence between 2006 and 2016. Furthermore, height and weight were measured by pediatric health professionals following a standard protocol across Catalonia.^19^ There are also limitations that should be considered when interpreting the results. The Information System for Research in Primary Care does not include information registered in primary care centers not pertaining to the Catalan Health Institute. However, the Catalan Health Institute is the main health provider in Catalonia, covering approximately 80% of the population, and SIDIAP has been shown to be highly representative of the Catalan region in terms of geographical area, age, and sex distributions.^16^ Also, SIDIAP is prone to participation bias, given that children with more severe illnesses might visit primary care more often and this could lead to an overestimation of prevalence and/or incidence rates. However, access to health care in Catalonia is universal, and height and weight are routinely measured by pediatric health professionals in primary care centers as part of the childhood with health program.^19^ The program recommends measuring length/height and weight several times during childhood and adolescence. Therefore, children visit the pediatrician even if they are healthy. Furthermore, no information on individual SES was available. The ecological deprivation index was based on data from the 2001 census, whereas the study period was from 2006 to 2016. However, it has been previously shown that SES does not change substantially between each census and period.^41^ Additionally, the deprivation index could only be estimated in urban areas, so we were not able to disentangle possible socioeconomic differences in rural areas.

## Conclusions

In this study, the overall prevalence and incidence rates of childhood overweight/obesity and obesity decreased during the last decade; however, we identified sociodemographic groups, including children living in the most deprived areas and with non-Spanish nationalities, among whom prevalence increased, giving rise to increasing deprivation disparities in childhood obesity. We also identified that children aged 6 and 7 years were most susceptible to developing overweight/obesity and obesity. Specific health initiatives, focusing on these groups, are urgently needed to tackle the alarmingly high prevalence of childhood overweight and obesity worldwide.

## Notes

Supplement. **eTable 1.** Prevalence Trend of Overweight/Obesity and Obesity by Sex, Age Category, and Urban/Rural Residence, 2006-2016 **eTable 2.** Prevalence Trend of Overweight/Obesity and Obesity by Sex, Age Category, and Nationality, 2006-2016 **eTable 3.** Incidence Rate (No. of New Cases per 100 Person-Years) of Overweight/Obesity and Obesity by Sex and Age, 2006-2016 **eTable 4.** Incidence Rate Ratios of Overweight/Obesity by Sex, Age, Deprivation Index, Urban/Rural Residence, Nationality, and Period, 2006-2016 **eTable 5.** Incidence Rate Ratios of Obesity by Sex, Age, Deprivation Index, Urban/Rural Residence, Nationality, and Period, 2006-2016 **eFigure 1.** Flow Chart of Studied Population **eFigure 2.** Prevalence Trends of Overweight/Obesity and Obesity by Sex and Age Category, 2006-2016 **eFigure 3.** Percentage Change in the Prevalence of Overweight/Obesity and Obesity by Sex, Age Category, and Deprivation Index, 2006-2016 **eFigure 4.** Prevalence Trends of Overweight/Obesity and Sex, Age Category, and Nationality, 2006-2016 **eFigure 5.** Obesity Incidence by Sex According to Their *z*BMI Category Classification at Baseline, 2006-2016
