# Supplementary material for: Time Trends and Sociodemographic Factors Associated With Overweight and Obesity in Children and Adolescents in Spain
Source: JAMA Netw Open. 2020 Mar 18;3(3):e201171. doi: 10.1001/jamanetworkopen.2020.1171 (PMC7081120; doi:10.1001/jamanetworkopen.2020.1171)
Supplement: Supplement. — eTable 1. Prevalence Trend of Overweight/Obesity and Obesity by Sex, Age Category, and Urban/Rural Residence, 2006-2016 eTable 2. Prevalence Trend of Overweight/Obesity and Obesity by Sex, Age Category, and Nationality, 2006-2016 eTable 3. Incidence Rate (No. of New Cases per 100 Person-Years) of Overweight/Obesity and Obesity by Sex and Age, 2006-2016 eTable 4. Incidence Rate Ratios of Overweight/Obesity by Sex, Age, Deprivation Index, Urban/Rural Residence, Nationality, and Period, 2006-2016 eTable 5. Incidence Rate Ratios of Obesity by Sex, Age, Deprivation Index, Urban/Rural Residence, Nationality, and Period, 2006-2016 eFigure 1. Flow Chart of Studied Population eFigure 2. Prevalence Trends of Overweight/Obesity and Obesity by Sex and Age Category, 2006-2016 eFigure 3. Percentage Change in the Prevalence of Overweight/Obesity and Obesity by Sex, Age Category, and Deprivation Index, 2006-2016 eFigure 4. Prevalence Trends of Overweight/Obesity and Sex, Age Category, and Nationality, 2006-2016 eFigure 5. Obesity Incidence by Sex According to Their zBMI Category Classification at Baseline, 2006-2016 [file jamanetwopen-3-e201171-s001.pdf]

Supplementary Online Content

de Bont J, Díaz Y, Casas M, García-Gil M, Vrijheid M, Duarte-Salles T. Time trends and sociodemographic factors associated with overweight and obesity in children and adolescents in Spain. *JAMA Netw Open*. 2020;3(3):e201171. doi:10.1001/jamanetworkopen.2020.1171

eTable 1. Prevalence Trend of Overweight/Obesity and Obesity by Sex, Age Category, and Urban/Rural Residence, 2006-2016

eTable 2. Prevalence Trend of Overweight/Obesity and Obesity by Sex, Age Category, and Nationality, 2006-2016

eTable 3. Incidence rate (No. of New Cases per 100 Person-Years) of Overweight/Obesity and Obesity by Sex and Age, 2006-2016

eTable 4. Incidence Rate Ratios of Overweight/Obesity by Sex, Age, Deprivation Index, Urban/Rural Residence, Nationality, and Period, 2006-2016

eTable 5. Incidence Rate Ratios of Obesity by Sex, Age, Deprivation Index, Urban/Rural Residence, Nationality, and Period, 2006-2016

eFigure 1. Flow Chart of Studied Population

eFigure 2. Prevalence Trends of Overweight/Obesity and Obesity by Sex and Age Category, 2006-2016

eFigure 3. Percentage Change in the Prevalence of Overweight/Obesity and Obesity by Sex, Age Category, and Deprivation Index, 2006-2016

eFigure 4. Prevalence Trends of Overweight/Obesity and Sex, Age Category, and Nationality, 2006-2016

eFigure 5. Obesity Incidence by Sex According to Their *z*BMI Category Classification at Baseline, 2006-2016

This supplementary material has been provided by the authors to give readers additional information about their work.

© 2020 de Bont J et al. *JAMA Network Open.*

eTable 1. Prevalence Trend of Overweight/Obesity and Obesity by Sex, Age Category, and Urban/Rural Residence, 2006-2016

			Overweight/obesity					Obesity			
		**Urban/ rural residence**	**Prevalence ratio 2006 (95% CI)[a]**	**Prevalence ratio 2016 (95% CI)[a]**	**Change from 2006 to 2016 (%)[b]**	**p-trend[c]**		**Prevalence ratio 2006 (95% CI)[a]**	**Prevalence ratio 2016 (95% CI)[a]**	**Change from 2006 to 2016 (%)[b]**	**p-trend[c]**
Boys, age											
	2-5 y	Rural	ref	ref	-26.27	<0.01		ref	ref	-28.95	<0.01
		Urban	1.16 (1.09-1.23)	1.22 (1.14-1.32)	-21.96	<0.01		1.21 (1.08-1.36)	1.40 (1.21-1.61)	-18.03	<0.01
	6-11 y	Rural	ref	ref	-8.54	<0.01		ref	ref	-7.28	<0.01
		Urban	1.09 (1.05-1.12)	1.14 (1.10-1.17)	-4.52	<0.01		1.17 (1.12-1.23)	1.23 (1.18-1.28)	-2.76	<0.01
	12-17 y	Rural	ref	ref	-9.07	<0.01		ref	ref	-8.39	<0.01
		Urban	1.10 (1.04-1.15)	1.16 (1.11-1.21)	-3.86	<0.01		1.15 (1.06-1.25)	1.16 (1.09-1.24)	-7.51	<0.01
Girls, age											
	2-5 y	Rural	ref	ref	-30.25	<0.01		ref	ref	-24.22	<0.01
		Urban	1.12 (1.05-1.19)	1.25 (1.16-1.36)	-21.70	<0.01		1.23 (1.08-1.40)	1.29 (1.11-1.51)	-20.62	<0.01
	6-11 y	Rural	ref	ref	-6.18	<0.01		ref	ref	-5.10	<0.01
		Urban	1.12 (1.08-1.16)	1.12 (1.09-1.16)	-5.62	<0.01		1.18 (1.11-1.25)	1.19 (1.13-1.25)	-4.61	0.05
	12-17 y	Rural	ref	ref	-3.49	<0.01		ref	ref	-7.60	0.11
		Urban	1.08 (1.02-1.14)	1.11 (1.07-1.17)	-0.61	<0.01		1.03 (0.94-1.14)	1.14 (1.05-1.24)	1.79	<0.01

[a] Prevalence ratios were calculated using a Poisson regression with robust variance.
[b] Change from 2006 to 2016 (%): (prevalence 2016 – prevalence 2006) / prevalence 2006 * 100.
[c] Logistic regression models were applied to test the p-trend using the study year as a continuous variable

© 2020 de Bont J et al. *JAMA Network Open.*

eTable 2. Prevalence Trend of Overweight/Obesity and Obesity by Sex, Age Category, and Nationality, 2006-2016

	Nationality	Overweight/obesity				Obesity			
		Prevalence ratio 2006 (95% CI)[a]	Prevalence ratio 2016 (95% CI)[a]	Change from 2006 to 2016 (%)[b]	p-trend[c]	Prevalence ratio 2006 (95% CI)[a]	Prevalence ratio 2016 (95% CI)[a]	Change from 2006 to 2016 (%)[b]	p-trend[c]
Boys, age									
2-5 y	Spanish	ref	ref	-25.95	<0.01	ref	ref	-25.51	<0.01
	African	0.81 (0.69-0.96)	1.17 (1.06-1.30)	7.43	0.85	0.60 (0.42-0.86)	1.20 (1.01-1.44)	49.31	0.02
	North/Central/South American	1.32 (1.08-1.61)	2.03 (1.72-2.39)	13.80	0.41	1.18 (0.80-1.74)	2.47 (1.87-3.25)	55.71	0.28
	Asian	0.89 (0.64-1.23)	1.39 (1.19-1.61)	15.26	0.45	0.89 (0.49-1.62)	1.80 (1.41-2.30)	50.28	0.78
	European	0.84 (0.68-1.03)	1.07 (0.90-1.27)	-5.54	0.14	0.70 (0.46-1.06)	1.17 (0.86-1.57)	24.54	0.72
6-11 y	Spanish	ref	ref	-6.05	<0.01	ref	ref	-4.46	<0.01
	African	0.48 (0.42-0.54)	0.79 (0.75-0.84)	55.22	<0.01	0.37 (0.30-0.46)	0.77 (0.71-0.83)	97.39	<0.01
	North/Central/South American	1.20 (1.11-1.30)	1.31 (1.21-1.41)	1.94	0.73	1.30 (1.16-1.45)	1.44 (1.29-1.59)	5.78	0.54
	Asian	0.62 (0.50-0.75)	1.15 (1.07-1.23)	75.47	<0.01	0.78 (0.60-1.01)	1.27 (1.15-1.39)	55.40	<0.01
	European	0.79 (0.71-0.88)	0.94 (0.88-1.01)	12.06	<0.01	0.70 (0.59-0.83)	0.85 (0.76-0.95)	16.51	0.02
12-17 y	Spanish	ref	ref	-5.29	<0.01	ref	ref	-8.41	<0.01
	African	0.45 (0.38-0.55)	0.58 (0.52-0.64)	20.32	<0.01	0.38 (0.27-0.53)	0.49 (0.41-0.60)	18.91	0.03
	North/Central/South American	1.14 (1.02-1.28)	1.17 (1.08-1.27)	-2.73	0.72	1.20 (1.00-1.43)	1.24 (1.09-1.42)	-4.96	0.13
	Asian	0.64 (0.50-0.82)	0.99 (0.88-1.10)	46.84	<0.01	0.61 (0.40-0.91)	1.07 (0.90-1.28)	62.55	<0.01
	European	0.88 (0.75-1.03)	1.02 (0.92-1.13)	9.69	0.66	0.86 (0.67-1.12)	1.08 (0.91-1.27)	14.06	0.92
Girls, age									
2-5 y	Spanish	ref	ref	-24.89	<0.01	ref	ref	-25.04	<0.01
	African	0.93 (0.79-1.09)	1.16 (1.05-1.29)	-6.09	0.03	1.01 (0.74-1.37)	1.25 (1.03-1.52)	-7.22	0.49
	North/Central/South American	1.69 (1.40-2.04)	1.84 (1.53-2.21)	-18.26	<0.01	1.98 (1.40-2.79)	2.53 (1.86-3.45)	-3.90	0.03
	Asian	0.79 (0.54-1.14)	0.94 (0.78-1.14)	-10.29	0.13	0.65 (0.29-1.45)	1.18 (0.84-1.65)	35.67	0.65
	European	0.86 (0.69-1.08)	1.04 (0.86-1.25)	-9.59	0.74	0.72 (0.44-1.15)	1.38 (1.01-1.90)	44.87	0.10
6-11 y	Spanish	ref	ref	-5.88	<0.01	ref	ref	-5.07	<0.01
	African	0.61 (0.54-0.69)	0.87 (0.83-0.92)	33.97	<0.01	0.56 (0.46-0.69)	0.85 (0.78-0.93)	43.60	<0.01
	North/Central/South American	1.15 (1.06-1.25)	1.26 (1.17-1.37)	3.43	0.95	1.22 (1.07-1.40)	1.41 (1.25-1.60)	9.69	0.29
	Asian	0.62 (0.50-0.79)	0.87 (0.80-0.95)	31.64	<0.01	0.39 (0.24-0.62)	0.80 (0.69-0.93)	95.76	<0.01
	European	0.79 (0.70-0.90)	0.90 (0.84-0.98)	7.06	<0.01	0.70 (0.56-0.86)	0.92 (0.81-1.04)	24.43	<0.01
12-17 y	Spanish	ref	ref	-2.42	<0.01	ref	ref	-2.31	<0.01
	African	0.85 (0.73-0.99)	1.05 (0.96-1.16)	20.50	0.05	0.66 (0.48-0.90)	1.15 (0.97-1.35)	70.78	0.02
	North/Central/South American	1.26 (1.12-1.41)	1.37 (1.26-1.49)	6.25	<0.01	1.29 (1.05-1.58)	1.44 (1.25-1.67)	9.37	0.02
	Asian	0.63 (0.46-0.87)	0.99 (0.86-1.13)	51.95	<0.01	0.49 (0.25-0.94)	1.13 (0.90-1.43)	125.76	<0.01
	European	0.99 (0.84-1.17)	0.99 (0.89-1.11)	-2.37	0.36	1.21 (0.92-1.58)	1.08 (0.88-1.31)	-12.94	0.42

[a] Prevalence ratios were calculated using a Poisson regression with robust variance.
[b] Change from 2006 to 2016 (%): (prevalence 2016 – prevalence 2006) / prevalence 2006 * 100.
[c] Logistic regression models were applied to test the p-trend using the study year as a continuous variable

© 2020 de Bont J et al. *JAMA Network Open.*

eTable 3. Incidence rate (No. of New Cases per 100 Person-Years) of Overweight/Obesity and Obesity by Sex and Age, 2006-2016

		**Incidence overweight/obesity**			**Incidence obesity**	
		**Cases**	**Nº new cases / 100 person-year (95% CI)**		**Cases**	**Incidence Nº new cases / 100 person-year (95% CI)**
Boys, age						
	2-3 y	6356	1.78 (1.74-1.83)		2043	0.54 (0.52-0.57)
	4-5 y	19442	4.49 (4.42-4.55)		8463	1.78 (1.74-1.81)
	6-7 y	40030	11.87 (11.75-11.99)		20800	4.86 (4.80-4.93)
	8-9 y	20699	7.86 (7.75-7.97)		14073	3.88 (3.82-3.95)
	10-11 y	10465	4.91 (4.81-5.00)		5913	1.89 (1.84-1.94)
	12-13 y	5480	3.10 (3.02-3.19)		2717	1.00 (0.97-1.04)
	14-15 y	2193	1.58 (1.51-1.64)		1083	0.50 (0.47-0.53)
	16-17 y	543	0.55 (0.50-0.60)		424	0.28 (0.25-0.31)
Girls, age						
	2-3 y	5584	1.67 (1.62-1.71)		1705	0.48 (0.46-0.50)
	4-5 y	16131	3.91 (3.85-3.97)		6578	1.45 (1.42-1.49)
	6-7 y	36729	11.23 (11.11-11.34)		15992	3.85 (3.79-3.91)
	8-9 y	18953	7.33 (7.23-7.44)		9637	2.65 (2.60-2.71)
	10-11 y	7698	3.54 (3.46-3.62)		3612	1.12 (1.08-1.15)
	12-13 y	4464	2.39 (2.32-2.46)		2011	0.71 (0.68-0.74)
	14-15 y	2316	1.52 (1.46-1.58)		955	0.41 (0.38-0.44)
	16-17 y	968	0.85 (0.80-0.91)		554	0.32 (0.30-0.35)

© 2020 de Bont J et al. *JAMA Network Open.*

eTable 4. Incidence Rate Ratios of Overweight/Obesity by Sex, Age, Deprivation Index, Urban/Rural Residence, Nationality, and Period, 2006-2016

Overweight/obesity		Incidence Rate Ratio (CI 95%) for each age categories							
		**2-3 y**	**4-5 y**	**6-7 y**	**8-9 y**	**10-11 y**	**12-13 y**	**14-15 y**	**16-17 y**
**Boys**									
Deprivation index	Least deprived	ref	ref	ref	ref	ref	ref	ref	ref
	Most deprived	1.35 (1.23-1.48)	1.18 (1.12-1.24)	1.21 (1.17-1.26)	1.21 (1.15-1.27)	1.20 (1.11-1.28)	1.09 (0.99-1.21)	1.11 (0.95-1.30)	0.77 (0.57-1.04)
Urban/rural residence	Rural	ref	ref	ref	ref	ref	ref	ref	ref
	Urban	1.07 (1.01-1.14)	1.10 (1.06-1.14)	1.13 (1.10-1.15)	1.14 (1.10-1.18)	1.07 (1.02-1.12)	1.08 (1.01-1.15)	1.02 (0.92-1.14)	0.93 (0.76-1.14)
Nationality	Spanish	ref	ref	ref	ref	ref	ref	ref	ref
	African	0.88 (0.79-0.99)	0.86 (0.80-0.91)	0.75 (0.72-0.79)	0.57 (0.52-0.61)	0.56 (0.50-0.63)	0.70 (0.60-0.81)	0.84 (0.68-1.05)	0.69 (0.42-1.13)
	American	1.98 (1.66-2.36)	1.45 (1.30-1.62)	1.23 (1.14-1.33)	1.17 (1.06-1.30)	1.16 (1.01-1.32)	1.17 (0.99-1.38)	1.01 (0.78-1.31)	1.60 (1.04-2.45)
	Asian	1.02 (0.84-1.23)	1.06 (0.95-1.17)	0.98 (0.91-1.05)	1.15 (1.04-1.27)	1.07 (0.92-1.24)	1.04 (0.85-1.27)	1.21 (0.91-1.61)	1.68 (1.02-2.75)
	European	0.88 (0.74-1.05)	0.79 (0.71-0.87)	0.78 (0.73-0.84)	0.81 (0.74-0.89)	0.88 (0.77-0.99)	1.07 (0.91-1.25)	1.08 (0.84-1.40)	2.26 (1.53-3.32)
Time periods	Period 1: (01/01/2006-30/06/2011)	ref	ref	ref	ref	ref	ref	ref	ref
	Period 2: (01/07/2011-31/12/2016)	0.80 (0.78-0.82)	0.94 (0.92-0.96)	0.95 (0.92-0.97)	0.95 (0.91-0.99)	1.05 (0.99-1.12)	0.92 (0.85-1.01)	1.33 (1.10-1.60)	0.80 (0.78-0.82)
**Girls**									
Deprivation index	Least deprived	ref	ref	ref	ref	ref	ref	ref	ref
	Most deprived	1.38 (1.25-1.53)	1.25 (1.18-1.33)	1.25 (1.20-1.30)	1.30 (1.23-1.37)	1.35 (1.24-1.47)	1.53 (1.37-1.71)	1.68 (1.44-1.95)	2.20 (1.75-2.78)
Urban/rural residence	Rural	ref	ref	ref	ref	ref	ref	ref	ref
	Urban	1.12 (1.05-1.20)	1.07 (1.03-1.11)	1.12 (1.10-1.15)	1.14 (1.10-1.18)	1.08 (1.03-1.15)	1.13 (1.05-1.21)	1.03 (0.93-1.14)	1.25 (1.06-1.48)
Nationality	Spanish	ref	ref	ref	ref	ref	ref	ref	ref
	African	0.89 (0.79-0.99)	0.78 (0.73-0.84)	0.81 (0.77-0.85)	0.78 (0.72-0.84)	0.94 (0.84-1.05)	1.57 (1.38-1.77)	2.13 (1.82-2.49)	2.35 (1.84-3.00)
	American	1.53 (1.24-1.89)	1.45 (1.29-1.63)	1.24 (1.14-1.33)	1.12 (1.01-1.24)	1.28 (1.12-1.48)	1.82 (1.58-2.10)	1.87 (1.56-2.26)	3.02 (2.41-3.79)
	Asian	0.98 (0.79-1.22)	0.71 (0.61-0.81)	0.73 (0.67-0.80)	0.83 (0.73-0.94)	1.20 (1.01-1.42)	1.40 (1.13-1.73)	1.53 (1.14-2.05)	1.63 (1.02-2.59)
	European	0.66 (0.54-0.82)	0.78 (0.70-0.87)	0.71 (0.66-0.77)	0.75 (0.68-0.83)	1.03 (0.90-1.18)	1.28 (1.08-1.51)	1.46 (1.17-1.83)	1.77 (1.27-2.46)
Time periods	Period 1: (01/01/2006-30/06/2011)	ref	ref	ref	ref	ref	ref	ref	ref
	Period 2: (01/07/2011-31/12/2016)	0.79 (0.76-0.81)	0.94 (0.92-0.96)	1.00 (0.97-1.03)	1.00 (0.95-1.05)	1.00 (0.93-1.07)	0.94 (0.86-1.03)	0.89 (0.78-1.02)	0.79 (0.76-0.81)

© 2020 de Bont J et al. *JAMA Network Open.*

eTable 5. Incidence Rate Ratios of Obesity by Sex, Age, Deprivation Index, Urban/Rural Residence, Nationality, and Period, 2006-2016

Obesity		Incidence Rate Ratio (CI 95%) for each age categories							
		**2-3 y**	**4-5 y**	**6-7 y**	**8-9 y**	**10-11 y**	**12-13 y**	**14-15 y**	**16-17 y**
**Boys**									
Deprivation index	Least deprived	ref	ref	ref	ref	ref	ref	ref	ref
	Most deprived	2.11 (1.77-2.51)	1.59 (1.46-1.72)	1.54 (1.46-1.62)	1.52 (1.42-1.62)	1.51 (1.37-1.67)	1.63 (1.41-1.88)	1.64 (1.30-2.08)	1.47 (1.03-2.09)
Urban/rural residence	Rural	ref	ref	ref	ref	ref	ref	ref	ref
	Urban	1.22 (1.09-1.36)	1.20 (1.14-1.27)	1.20 (1.16-1.24)	1.23 (1.18-1.28)	1.14 (1.07-1.21)	1.20 (1.09-1.32)	1.22 (1.05-1.43)	1.17 (0.91-1.50)
Nationality	Spanish	ref	ref	ref	ref	ref	ref	ref	ref
	African	0.99 (0.82-1.19)	0.80 (0.72-0.88)	0.75 (0.70-0.81)	0.55 (0.49-0.61)	0.57 (0.48-0.67)	0.63 (0.49-0.81)	0.69 (0.48-1.01)	1.00 (0.59-1.71)
	American	2.55 (1.93-3.36)	1.63 (1.39-1.91)	1.27 (1.15-1.41)	1.27 (1.13-1.43)	1.35 (1.16-1.59)	1.63 (1.34-1.98)	1.61 (1.20-2.15)	1.49 (0.90-2.45)
	Asian	1.88 (1.45-2.43)	1.42 (1.24-1.63)	1.12 (1.02-1.24)	1.22 (1.08-1.38)	1.13 (0.93-1.39)	1.43 (1.10-1.86)	1.21 (0.78-1.88)	2.64 (1.60-4.34)
	European	0.91 (0.67-1.24)	0.78 (0.66-0.91)	0.83 (0.76-0.92)	0.79 (0.70-0.89)	0.93 (0.79-1.11)	1.18 (0.94-1.48)	1.03 (0.69-1.53)	2.98 (1.99-4.47)
Time periods	Period 1: (01/01/2006-30/06/2011)	ref	ref	ref	ref	ref	ref	ref	ref
	Period 2: (01/07/2011-31/12/2016)	0.86 (0.82-0.90)	0.92 (0.89-0.95)	0.94 (0.91-0.98)	0.99 (0.93-1.05)	1.06 (0.97-1.16)	1.03 (0.91-1.16)	0.94 (0.77-1.15)	0.86 (0.82-0.90)
**Girls**									
Deprivation index	Least deprived	ref	ref	ref	ref	ref	ref	ref	ref
	Most deprived	1.90 (1.57-2.30)	1.65 (1.50-1.81)	1.68 (1.58-1.79)	1.88 (1.74-2.04)	1.82 (1.60-2.07)	1.92 (1.62-2.27)	2.32 (1.80-2.99)	3.79 (2.66-5.38)
Urban/rural residence	Rural	ref	ref	ref	ref	ref	ref	ref	ref
	Urban	1.22 (1.07-1.38)	1.14 (1.07-1.21)	1.19 (1.14-1.23)	1.10 (1.04-1.15)	1.11 (1.02-1.20)	1.25 (1.12-1.41)	0.99 (0.85-1.16)	1.19 (0.96-1.49)
Nationality	Spanish	ref	ref	ref	ref	ref	ref	ref	ref
	African	0.96 (0.78-1.17)	0.71 (0.63-0.80)	0.83 (0.77-0.89)	0.77 (0.69-0.87)	1.01 (0.85-1.20)	1.65 (1.36-2.01)	2.43 (1.90-3.10)	2.36 (1.69-3.30)
	American	2.11 (1.52-2.92)	1.78 (1.51-2.10)	1.34 (1.19-1.49)	1.28 (1.12-1.46)	1.42 (1.17-1.71)	2.33 (1.94-2.81)	1.83 (1.38-2.42)	2.69 (1.99-3.63)
	Asian	0.75 (0.48-1.18)	0.81 (0.65-1.00)	0.66 (0.56-0.77)	0.71 (0.58-0.87)	1.26 (0.97-1.64)	2.07 (1.56-2.76)	2.19 (1.45-3.31)	2.48 (1.46-4.21)
	European	1.10 (0.81-1.50)	0.81 (0.68-0.96)	0.82 (0.73-0.91)	0.67 (0.58-0.79)	1.25 (1.03-1.52)	1.30 (1.00-1.70)	1.58 (1.11-2.24)	1.07 (0.60-1.90)
Time periods	Period 1: (01/01/2006-30/06/2011)	ref	ref	ref	ref	ref	ref	ref	ref
	Period 2: (01/07/2011-31/12/2016)	0.86 (0.81-0.90)	0.94 (0.91-0.97)	0.97 (0.93-1.02)	0.95 (0.88-1.02)	1.08 (0.98-1.19)	1.00 (0.88-1.15)	0.95 (0.80-1.14)	0.86 (0.81-0.90)

© 2020 de Bont J et al. *JAMA Network Open.*

eFigure 1. Flow Chart of Studied Population

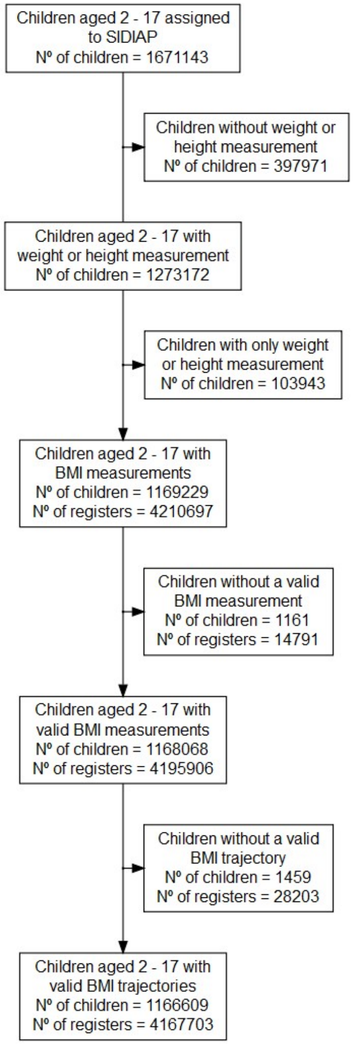

© 2020 de Bont J et al. *JAMA Network Open.*

eFigure 2. Prevalence Trends of Overweight /Obesity and Obesity by Sex and Age Category, 2006-2016

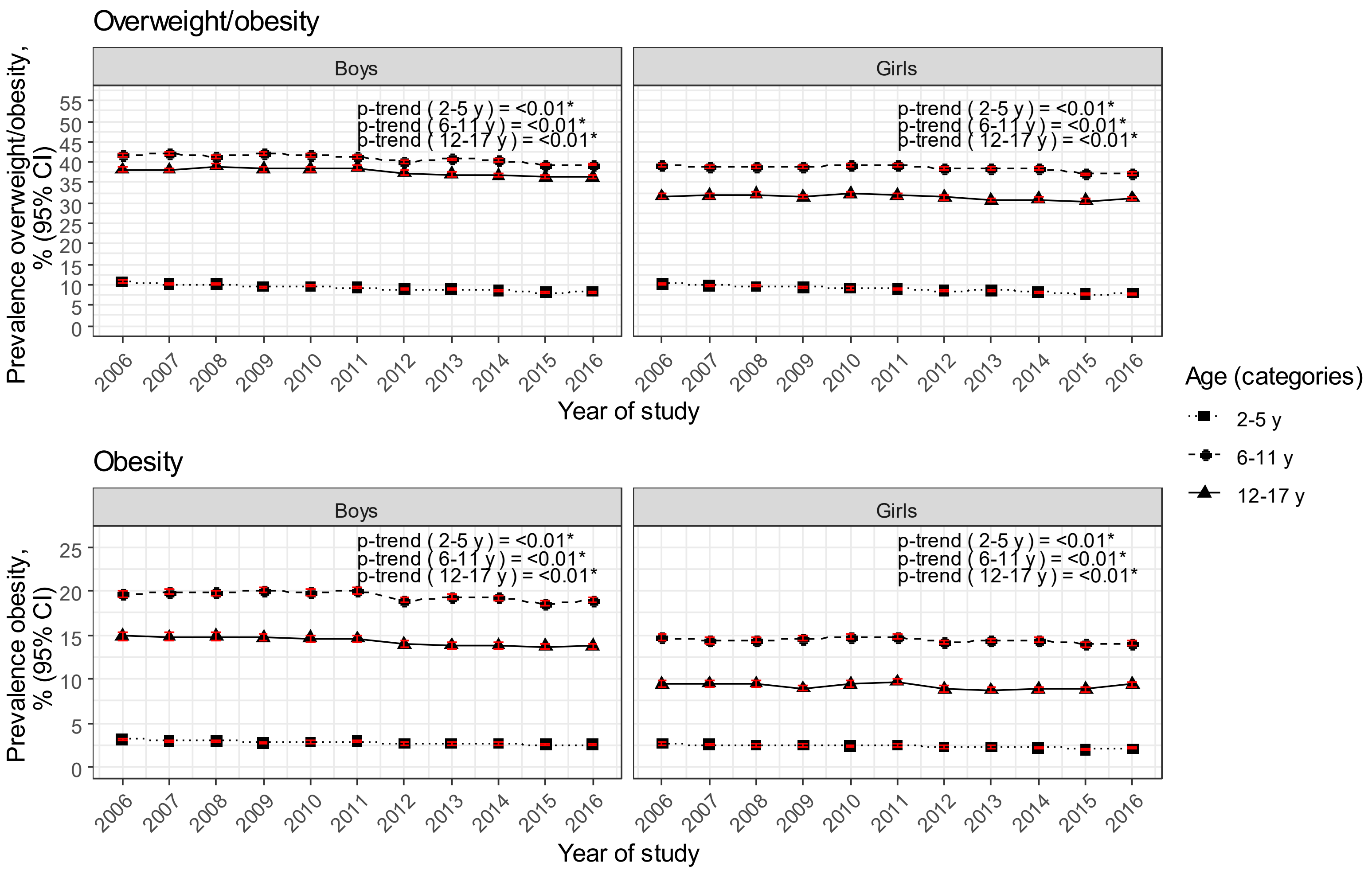

Prevalence trends over time were evaluated with a logistic regression models using the study year as a continuous variable (independent variable) and overweight and obesity as binary variables (dependent variables).

© 2020 de Bont J et al. *JAMA Network Open.*

eFigure 3. Percentage Change in the Prevalence of Overweight/Obesity and Obesity by Sex, Age Category, and Deprivation Index, 2006-2016

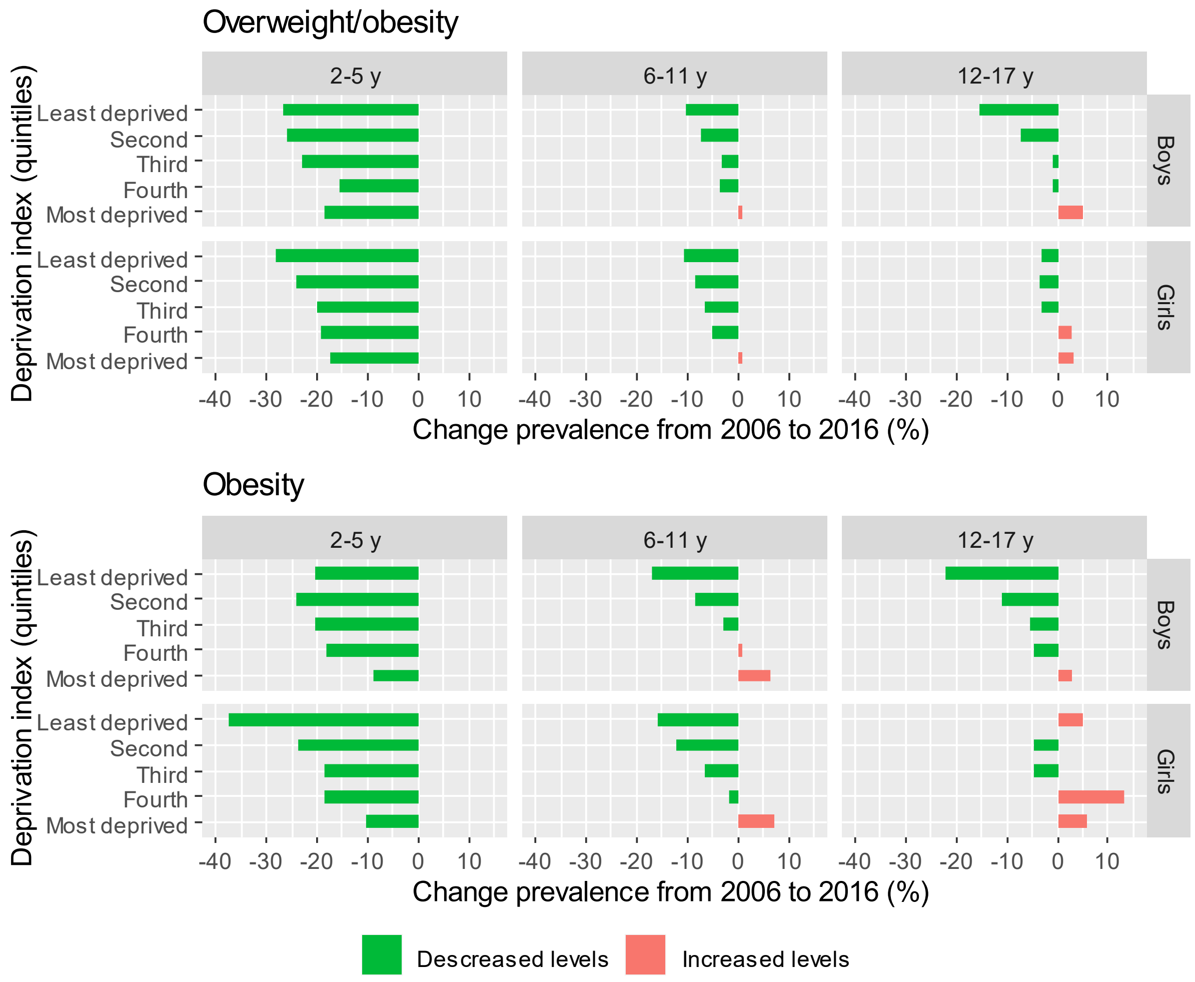

Change from 2006 to 2016 (%) by deprivation index: (prevalence 2016 – prevalence 2006) / prevalence 2006 * 100.

© 2020 de Bont J et al. *JAMA Network Open.*

eFigure 4. Prevalence Trends of Overweight/Obesity and Sex, Age Category, and Nationality, 2006-2016

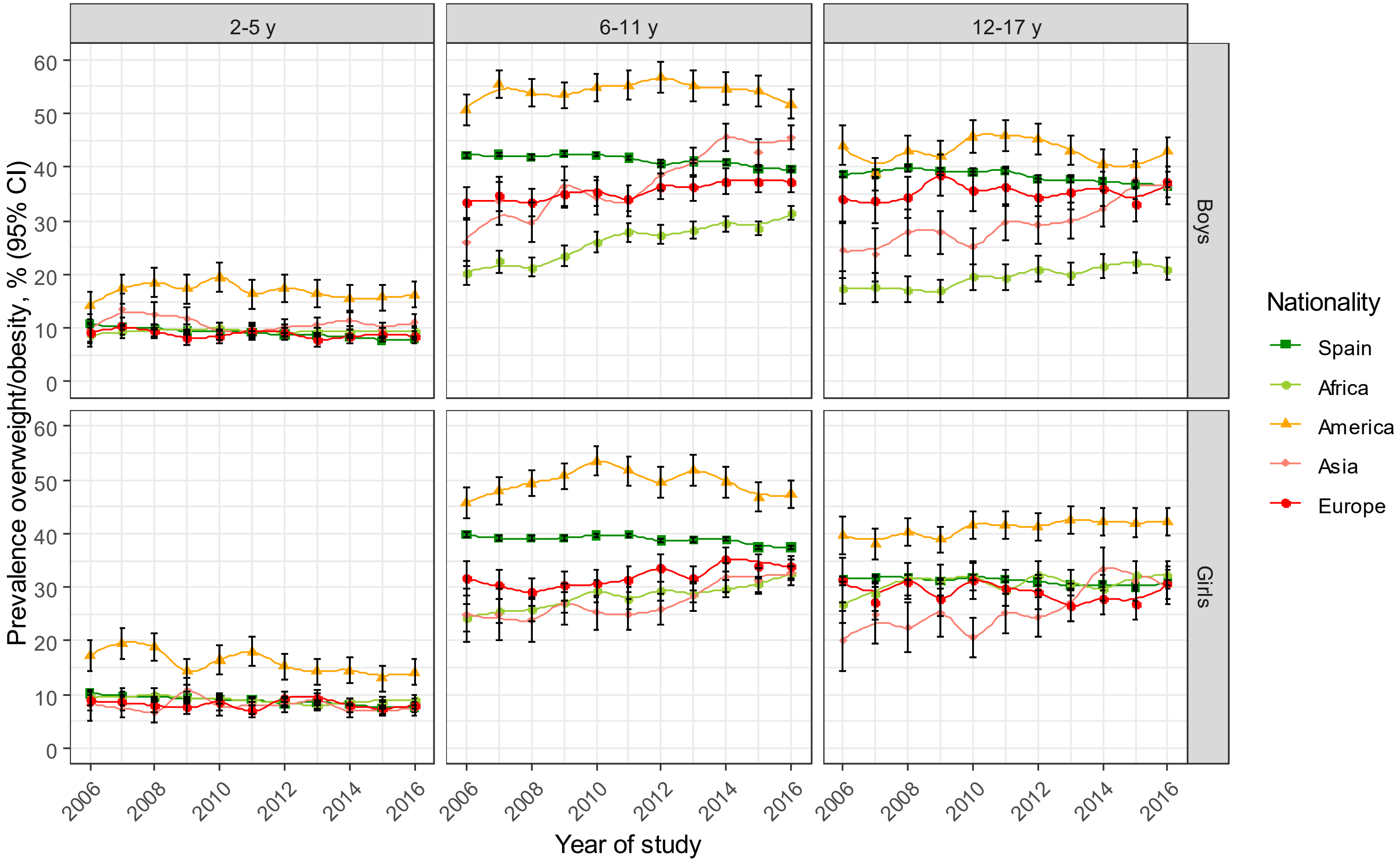

Note: prevalence trends of overweight/obesity with 95% CIs (black error bars) stratified by sex, age category and nationality

© 2020 de Bont J et al. *JAMA Network Open.*

eFigure 5. Obesity Incidence by Sex According to Their *z*BMI Category Classification at Baseline, 2006-2016

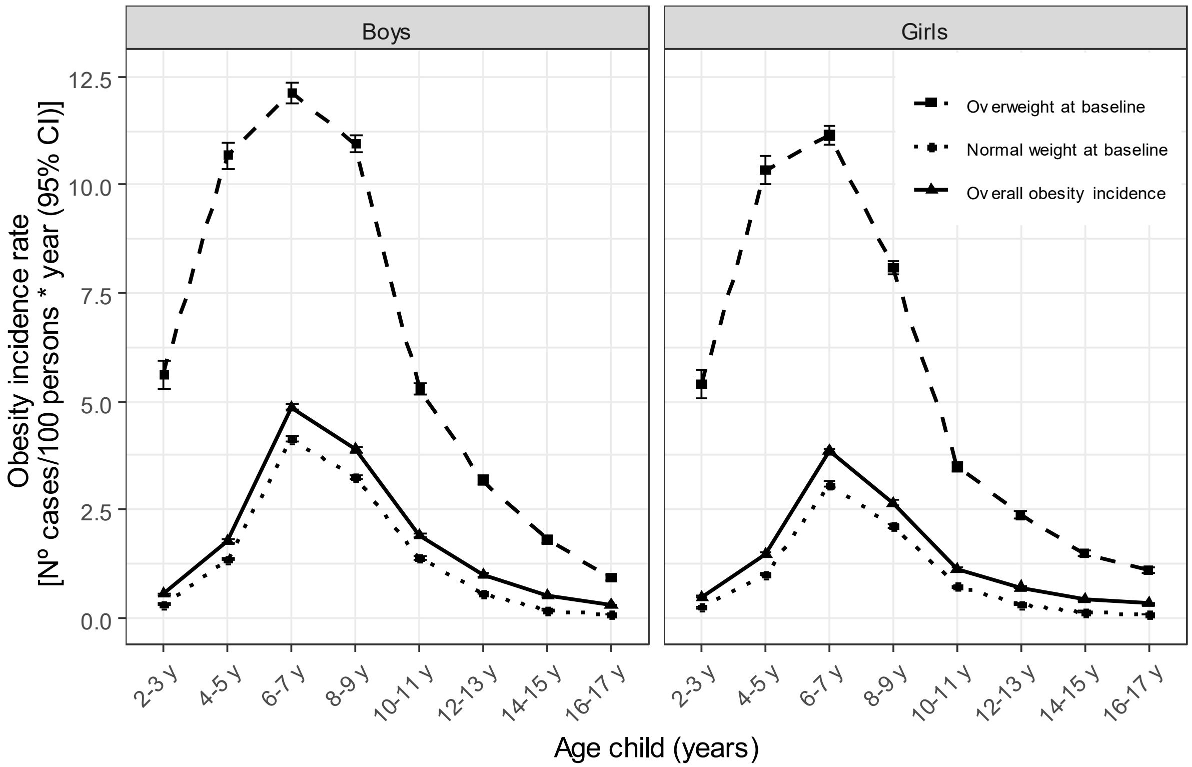

Overweight and normal weight at baseline was children who were overweight and normal weight at their first BMI measurement, respectively. Overall obesity incidence is the incidence of both children who were overweight or normal weight at baseline.

© 2020 de Bont J et al. *JAMA Network Open.*
